# New insights into the phenomenon of remissions and relapses in autoimmune diseases and the puzzle of benign autoantibodies in healthy individuals

**DOI:** 10.3389/fimmu.2025.1522356

**Published:** 2025-05-09

**Authors:** Zeev Elkoshi

**Affiliations:** Research and Development Department, Taro Pharmaceutical Industries Ltd, Haifa, Israel

**Keywords:** autoimmune diseases, remission, relapse, tissue recovery rate, autoantibodies, immunosuppressants

## Abstract

The onset and relapse of autoimmune diseases (AIDs) are triggered by autoimmune attacks on target tissues. However, symptoms are unlikely to appear if damaged cells are rapidly replaced. Addressing the implications of this premise, the present work examines the balance between target tissue destruction and recovery rates as a key factor in the mechanisms of remissions and relapses in AIDs. The theory, supported by published clinical data, suggests that remissions are improbable in AIDs characterized by slow target tissue recovery. Conversely, a high recovery rate is a necessary (though not sufficient) condition for cycles of remission and relapse in AIDs. A high recovery rate of target tissue explains the tendency for remitting-relapsing disease, the likelihood of detecting autoantibodies in healthy individuals and the responsiveness to immunosuppressive drug treatments. Analyzing specific AIDs through the balance of tissue destruction and recovery yields several insights. For example, the difference between androgenic alopecia, a non-remitting-relapsing disease and alopecia areata, a remitting-relapsing AID, is elucidated. A new mechanism underlying relapses and remissions in alopecia areata based on hair follicle regeneration rate is proposed. It is suggested that mild Graves’ disease and remitting Hashimoto’s thyroiditis would be responsive to corticosteroids or immunosuppressant treatment, unlike more severe forms of these diseases. Additionally, it is proposed that the transition from remitting-relapsing multiple sclerosis to secondary progressive multiple sclerosis is associated with the depletion of brain compensatory reserves. Notably, it is concluded that exercise will not play a neuroprotective role in secondary progressive multiple sclerosis.

## Introduction

Relapses refer to periods when a disease’s severity intensifies, while remissions are periods when its severity diminishes. Many autoimmune diseases (AIDs) are characterized by alternating phases of relapse and remission, with symptoms flaring up and then subsiding. Conditions following this pattern include multiple sclerosis (MS), rheumatoid arthritis, inflammatory bowel disease, lupus, myasthenia gravis, and psoriasis (1). These cycles of remission and relapse are driven by fluctuating immune reaction that sways between pro- and anti-inflammatory responses. However, in certain AIDs other factors may be involved in remission (MS for example). The onset and relapse of AIDs may be triggered by internal factors, such as epitope spreading, reduced immune regulation, and an increased inflammatory response (2). External factors such as viral infections, low exposure to sunlight (3) and stress (4) can also trigger the onset and relapse of AIDs. Remission may occur when inflammation subsides, a viral infection resolves, or stress is alleviated. While immune system activity is often proposed to underlie the cycles of flare and abatement in AIDs (1, 2, 5), the role of target tissue recovery rate is less commonly discussed as a factor in the onset, relapse, and remission of AIDs. An earlier publication on the refractoriness of certain AIDs to immunosuppressants suggests that the balance between target tissue destruction and recovery rates, either during an autoimmune attack or following immunosuppressive treatment, is crucial for determining tissue recovery and symptom improvement (6). The present article adopts a similar approach to explore remissions and relapses in AIDs and addresses the puzzle of harmless autoantibodies in healthy individuals.

## Remissions and relapses in autoimmune diseases: understanding their onset and termination

Define the net tissue generation rate during immune attack, *R*, as:


(1) R=Rreg−Rdes

where *Rreg* is the regeneration rate of the tissue under the autoimmune attack, and *Rdes* is the destruction rate of the tissue due to the autoimmune attack. When *R* > 0, the autoimmune response is unlikely to trigger symptoms, as the regeneration rate exceeds the destruction rate, thereby preserving tissue mass during the immune attack. Symptoms of AIDs appear when *R* **<** 0 which occurs when *Rreg* **<** *Rdes*. Under this condition, continued immune attack will ultimately destroy all tissue cells. In such cases, spontaneous remission is unlikely, as any newly regenerated cells will be damaged by the ongoing immune response, preventing the tissue recovery necessary for symptom relief and remission. Clinical improvement (remission) is a consequence of tissue recovery which can take place, only when inflammation has sufficiently dampened, resulting in *R* > 0. This may happen spontaneously or following drug treatment. Without remission, a relapse is improbable, establishing a monophasic disease. Thus, a necessary condition for tissue remission (and a possible subsequent relapse) in AIDs is *R* > 0. Immunosuppressive drug treatments may lower the *Rdes* value, thereby inducing remission (*R* > 0).

If the tissue has an inherently negligible regeneration rate (*Rreg* = 0), *R* will be negative, even with a low destruction rate (*Rdes*). Consequently, tissues with negligible regeneration rates are unlikely to recover, making remission unlikely.

In certain tissues targeted by autoimmune attacks, variations in cell function also influence the likelihood of remission or relapse. For instance, fluctuations in insulin secretion rates by pancreatic β cells in Type 1 Diabetes (T1D) or changes in thyroid hormone secretion by thyroid follicles in Hashimoto’s Thyroiditis (HT) can trigger symptom remission or relapse. The impact of these cells on remission or relapse depends not only on their quantity but also on the rate at which they produce and release hormones. In other AIDs, such as MS, functional impairment caused by immune reaction is a more appropriate measure for relapses and remissions. The term “tissue activity” will be used to describe target tissue function. It is convenient to define *Ra*, the net rate of tissue activity during an autoimmune attack, as:


(2) Ra=(Ra+)−(Ra−)

where *Ra+* represents the increase in *Ra* by factors like tissue regeneration or activating agents, while *Ra-* indicates the decrease in *Ra* due to tissue destruction following autoantibody attack, or from impairment of cell function by deactivating hormones or toxic substances. Throughout this text *Ra+* will be referred to as “recovery rate”. Remission is expected when *Ra* > 0, while relapse is expected when *Ra* **<** 0.

Equation 2 can be viewed as a generalization of Equation 1, as the net tissue generation rate is one of the factors that contribute to the overall rate of tissue activity. A prerequisite for applying Equation 2 is the presence of a minimum number of active cells in the target tissue; if the targeted tissue experiences significant damage, no activity is expected

Here are five examples of chronic diseases (four AIDs and one non-AID) that typically do not go into remission in most patients:

### Type 1 diabetes (advanced)

T1D is the most common chronic AID in young patients. It is caused by the destruction of pancreatic endocrine β-cells, which are responsible for producing insulin in the islets of Langerhans. As a result, the body becomes deficient in insulin and experiences hyperglycemia. The primary treatment for T1D involves regular insulin injections to control hyperglycemia. A defining feature of T1D is the immune system’s recognition of β-cell proteins as autoantigens, leading to an autoimmune response involving autoreactive CD4+ and CD8+ T cells, as well as autoantibodies (7). The regenerative capacity of the pancreatic endocrine islets including that of β cells is limited in adults (8). In newly diagnosed T1D patients, many experience a decrease in insulin requirements after starting insulin therapy. This phase is known as the ‘honeymoon period.’ A study on new-onset T1D patients found that during the first 18 months of the disease, 12.3% of patients entered total remission (median duration of 6 months), and 18.3% experienced partial remission (median duration of 6 months). However, the percentage of patients in remission decreased after 6 months (9). This topic will be explored in greater detail later. Nevertheless, remissions in advanced T1D, after most β-cells have been destroyed, are extremely rare since almost all T1D patients require lifelong insulin replacement therapy (10). The author is not aware of any publications describing remission (i.e., no need for insulin replacement therapy) in advanced T1D after the honeymoon period has ended. The slow recovery rate in advanced T1D likely underlies this monophasic pattern.

### Androgenic alopecia

Pattern or androgenetic alopecia (AGA) is a hereditary condition caused by an increased sensitivity to androgens, affecting about 50% of both men and women. AGA is not an AID. It is marked by the gradual thinning of terminal hair on the scalp, usually beginning after puberty and following a characteristic pattern unique to each gender. Activation of androgen receptors shortens the anagen (growth) phase of the hair cycle, leading to follicular miniaturization. As a result, hair follicles become progressively finer and shorter, sometimes failing to grow long enough to reach the skin’s surface (11). Androgens act through the intracellular androgen receptor (AR). In hair follicles, ARs are mainly expressed by the dermal papilla (12–14). The prevailing hypothesis proposes that the androgen/AR complex modifies the production of regulatory factors within the dermal papilla, affecting both its own growth and the growth of the follicle’s epithelial components through autocrine and paracrine signaling (15). For example, stimulation of TGF-β production in HF papilla cells from AGA patients inhibited normal epithelial cells growth *in vitro* (16). AGA is a chronic and progressive disease. AGA entails inflammatory characteristics, at least in part of the patients (17–20). Regeneration of damaged hair follicle epithelial cells is impaired in AGA patients. Androgens disrupt the balance between Wnt agonists and antagonists in dermal papilla cells, inhibiting the differentiation of hair follicle stem cells (21). In addition, a defective conversion of HF stem cells to progenitor cells was reported in AGA (22). Since AGA is a chronic disease that involves progressive destruction of a target tissue (hair follicle), Eq. 1 may be applied to assess the likelihood of remission, although AGA is not an autoimmune condition. The low rate of scalp HF regeneration in AGA implies that remissions are not expected (*R* **<** 0). To the best of the author’s knowledge, no spontaneous remissions of AGA are reported in the literature.

### Hashimoto’s thyroiditis

The thyroid gland is characterized by its slow cell turnover rate, with cells estimated to divide approximately once every 10 years and around 5 times during adulthood (23, 24). Although recent findings indicate an increase in thyroid volume following hemithyroidectomy (used to treat goiter) due to epithelial hyperplasia, this compensatory mechanism has minimal or no impact on symptomatology (25). The destruction of thyrocytes by autoreactive T cells and the low regeneration capacity of the thyroid gland leads, over time, to an extensive destruction of thyroid follicular cells in many patients with Hashimoto’s thyroiditis (HT) (26). However, remissions are reported in some HT patients. Takasu et al. (27) reported that among a group of 92 HT patients treated with levothyroxine, 22 patients (24%) experienced remission after the treatment was stopped. In this study, patients in remission were defined as those who remained euthyroid for periods ranging from 1 to 8 years after treatment cessation. In another study, 8.1 years follow-up of children with HT revealed that over the years, 16% of children presenting with overt hypothyroidism stopped therapy (28). The present study suggests that increased production of thyroid hormones by a cluster of active thyroid follicles surviving the immune attack may contribute to symptom improvement in HT patients experiencing remission.

### Graves’ disease

Graves’ disease (GD) is an autoimmune disorder that primarily targets the thyroid gland and is the leading cause of hyperthyroidism. In GD, hyperthyroidism is caused by autoantibodies targeting the thyroid stimulating hormone receptor (TSHR), which act as agonists to stimulate excessive thyroid hormone production (29). Although spontaneous recovery from GD is rare, it has been reported in stress-induced GD cases following relief from stress (4). Remissions have also been observed in some GD patients after treatment with propranolol (30). Despite the very limited regenerative capacity of thyrocytes, existing thyroid cells can respond to external stimuli—such as TSH, glucocorticoids, catecholamines, and pro-inflammatory cytokines—by adjusting thyroid hormone secretion. At the same time, the active thyroid hormone T3 is degraded by iodothyronine deiodinases (31, 32). The balance between T3 production (stimulated by TSH and by TSHR stimulating autoantibodies and suppressed by TSHR blocking autoantibodies) and T3 degradation, dictates whether hypothyroidism in GD progresses or resolves. Enzymatic degradation of T3, as with any enzymatic process, reaches a maximum steady-state value (a plateau) at high substrate concentrations, in line with the Michaelis-Menten equation (33). When substrate concentration exceeds the level required to reach this plateau, the negative feedback mechanism is lost, meaning an increase in T3 generation rate is no longer counteracted by a rise in its degradation rate (as the enzymatic degradation rate remains constant). This explains the onset of GD symptoms when *Ra* > 0. GD remission occurs when *Ra* **<** 0. However, in cases of severe GD with very high T3 production, a moderate reduction in T3 generation rate (for example, following drug treatment or stress reduction in stress-induced GD) may not be sufficient to shift the balance and achieve remission. Therefore, severe GD does not respond to drugs, such as propranolol, that only moderately reduce T3 production, and ‘spontaneous’ remissions of GD are expected only in mild cases. Indeed, the remissions reported by Willems et al. (4) and Codaccioni et al. (30) involved only patients with mild GD. Remissions in patients treated with propranolol were observed in a subgroup with significantly lower T3 levels compared to those without remissions (30). Similarly, none of the patients with stress-induced GD exhibited thyroid eye disease or a large goiter (4), the classical signs of severe GD. The author of this article postulates that, in contrast to severe GD (6), patients with mild GD may benefit from immunosuppressive treatment, including corticosteroids.

### Primary biliary cholangitis

Primary biliary cholangitis (PBC) is a long-term AID primarily seen in middle-aged women, characterized by cholestasis. The condition involves the gradual destruction of intrahepatic bile ducts and the presence of circulating anti-mitochondrial antibodies. In severe cases, PBC can progress to complications such as fibrosis, cirrhosis, end-stage liver disease, and even death. The impairment of the Cl−/HCO3− exchanger AE2 in cholangiocytes and lymphoid cells leads to biliary epithelial cell (BEC) destruction, and in severe cases, to the destruction of hepatocytes as well and an impairment of liver regenerative capacity (6). Indeed, spontaneous or treatment-related PBC remissions are very rare (34).

In contrast to the five aforementioned examples, AIDs targeting tissues with a high recovery rate after damage, are generally more prone to remissions, which may or may not be followed by exacerbations. More specifically, a high recovery rate of the target tissue is a necessary (but not sufficient) condition for the remission of an autoimmune disease.

Here are five examples of autoimmune diseases prone to remissions and relapses:

### Type 1 diabetes (early disease)

T1D clinical onset with classic hyperglycemic symptoms occurs when less than 30% of β-cells remain. As mentioned above, shortly after T1D diagnosis, many patients enter a partial remission known as the “honeymoon phase,” lasting a few months. During this time, the remaining β-cells produce enough insulin to reduce the need for external insulin. However, this phase is temporary, and all patients eventually relapse, requiring lifelong insulin therapy (35). The availability of a considerable number of *active* β-cells shortly after T1D onset allows the remission period observed in many patients.

### Alopecia areata

Hair follicles (HFs) in the anagen phase of the hair cycle have a high regeneration rate (36). Alopecia areata (AA) is a form of non-scarring hair loss in humans, characterized by the collapse of hair follicle immune privilege and the infiltration of lymphocyte around the anagen bulb (37). HF stem cells are spared during AA (38, 39). The author of this work believes that, in contrast to AGA, stem cell activity is also preserved during AA, which secures the high regeneration rate of HFs. Indeed, remissions and relapses in alopecia areata are common (40). Immune privilege (IP) of anagen hair follicles is a unique characteristic that protects follicles from immune attacks. HF-IP effectively suppresses the immune cell response by establishing an immunosuppressive signaling environment in anagen HFs. Both stress and viral infections can trigger HF-IP collapse (41), mediated by immune cell secretion of IFN-γ (37, 42, 43). Spontaneous restoration of HF-IP is promoted by the intrafollicular production of endogenous IP guardians and the release of immunoinhibitory neuropeptides (37). Breitkopf et al. (44) reported the upregulation of somatostatin in the middle (sheath) and lower (bulb) portions of human HF. These authors have shown that PBMCs, cultured with stimulatory allogeneic epidermal cells and somatostatin, secreted significantly less IFN- γ than controls, suggesting somatostatin as a secretory factor potentially contributing to the HF-IP repertoire. Ito et al. (45) identified α-melanocyte stimulating hormone (α-MSH), insulin growth factor-1 (IGF-1), and tumor growth factor β1 (TGF-β1), all of which are locally generated in the skin, as factors that may potentially restore HF-IP following its collapse. These studies collectively suggest that the collapse and subsequent restoration of immune privilege drive the relapse-remission cycles in AA. The present study proposes an alternative mechanism, potentially operating alongside immune privilege modulation, in driving the cyclic pattern observed in AA. Qin et al. (46) demonstrated that IFN-γ inhibits the differentiation of adult liver and bone marrow hematopoietic stem cells in mice by blocking Notch signaling activation. The Notch signaling pathway is crucial for maintaining hair follicle growth and development (47). It is therefore plausible that IFN-γ similarly inhibits hair follicle stem cell proliferation through Notch signaling suppression, potentially preventing remission in AA while allowing relapse. Conversely, IGF-1, identified as an HF-IP promoter, also enhances mesenchymal stem cell self-renewal and proliferation (48) and may similarly support hair follicle stem cell proliferation. This would facilitate hair follicle cell regeneration. According to the premises of this study, a faster follicular cell regeneration rate could prevent relapses while enabling remissions.

It can be concluded that a balance between IFN-γ (secreted by activated T cells and NK cells) and the endogenous IP guardians, determines whether hair sheds or grows during AA. This balance can influence hair growth either through its effect on the follicular immune barrier or by affecting the rate of follicular cell regeneration.

In terms of Equation 1, *R*, the net rate of hair growth, is determined by the difference between *Rreg* (the hair growth rate) and *Rdes* (the hair destruction rate). Hair growth is promoted by agents that support HF-IP such as somatotropin, α-MSH, IGF-1, and TGF-β1 or by those that stimulate HF cells regeneration rate, like α-MSH. Hair destruction is driven by agents that either disrupt anagen HF-IP or slow down HF cell regeneration rates, such as IFN-γ in both cases. When *R* > 0, the anagen phase continues and hair grows. When *R* **<** 0, HFs experience dystrophy, transitioning into the catagen and telogen phases, which halts hair growth and leads to hair shaft breakage and shedding. Remission in AA may occur with occasional increases in local concentrations of IP-preserving agents or those that enhance regeneration rates. Relapse may occur with a decrease in these agents or an increase in IFN-γ concentration (due to stress or viral infection). Notably, without a consistently high regeneration rate of anagen HFs in AA, remissions and subsequent relapses would be unlikely.

### Autoimmune pancreatitis

As has been asserted, the regenerative capacity of the pancreas endocrine islets in adults is limited. In contrast, the exocrine pancreas can regenerate and fully recover from conditions like acute pancreatitis (8). Autoimmune pancreatitis (AIP) is an inflammatory disease that primarily affect the exocrine pancreas (49). Indeed, 80% spontaneous remission rate was reported in a group of 95 patients with untreated Type 1 autoimmune pancreatitis (pancreatic manifestation of IgG4-related disease) (50). Moreover, negative IgG4 staining of the duodenal papilla was the only independent predictor of spontaneous remission in AIP, whereas seropositivity was significantly associated with relapse (51). Since IgG4 is an antibody specifically associated with Type 1 AIP, these findings suggest that remission occurs when this specific immune response subsides, and relapse occurs when it reemerges. In other words, in Type 1 AIP, remissions and relapses are determined by changes in *Rdes* (Eq. 1), while *Rreg* remains relatively constant. It seems that AIP remission is induced by a reduction in immune response, and it is not surprising that corticosteroids are effective in ameliorating AIP (50). But this beneficial effect would not be observed if the regeneration rate of the exocrine pancreas was low.

### Inflammatory bowel diseases

The intestinal epithelium is typically renewed every 4**–**5 days, making it one of the fastest-regenerating tissues in the human body (52). As a result, inflammatory bowel diseases (IBD) are expected to follow a pattern of relapse and remission. Indeed, IBD progress with alternating phases of remission and flare-ups (53). Given the high turnover rate of the intestinal epithelium, these cycles are largely influenced by the degree of bowel inflammation. In fact, an increase in inflammatory markers such as fecal calprotectin can predict relapses, with a pooled sensitivity of 0.720 and specificity of 0.740 (53). If epithelial turnover were low (low *Rreg* value), a detection method with a lower limit of detection would be required to predict relapses, as even minimal levels of inflammatory markers could result in *R* **<** 0. The high renewal capacity of the intestines allows a large proportion of IBD patients to have mucosal inflammation without clinical symptoms (54).

### Multiple sclerosis

Multiple sclerosis is an inflammatory disease of the central nervous system (CNS) characterized by both inflammatory and degenerative processes that impact both white and grey matter. The key neuropathological features of MS include demyelination, inflammation, astrocytic gliosis, and neurodegeneration, with neurodegeneration now recognized as the primary pathological driver of clinical progression (55). Multiple sclerosis is considered an AID. Although there is no consensus, myelin-related antigens are suspected to play a role, with some studies also suggesting that antigens on the surfaces of neuronal or glial cells may be involved (3). Eighty percent of MS patients exhibit a pattern known as relapsing-remitting MS (RRMS), characterized by cycles of exacerbation followed by substantial remission. Relapses typically occur every few years, with symptoms—such as visual, motor, sensory, or autonomic disturbances affecting bowel and bladder function—depending on the location of the inflammatory attack in the CNS. These relapses can last days or weeks and are followed by remission, though some residual disability often remains. Over time, about one-third of patients with relapsing-remitting MS experience increasing disability, while the frequency of relapses and remissions diminishes, contrary to reasoning. At the point where relapse and remission wane, the condition is termed secondary-progressive MS (SPMS) (56). Applying Eq. 2 to RRMS, *Ra* is the patient’s motor function, *Ra-* is comprised of all factors that impair motor function while *Ra+* encompasses all factors that improve it. In this respect, *Ra-* includes rates of nerve demyelination and axonal injury due to immune attack. *Ra+* includes nerve remyelination rate, restoration rate of axonal function and brain’s compensatory effects reflected by an increased cortical response to nerve injury (57, 58). A relapse is observed when *Ra* **<** 0 (*Ra+* ***<*** *Ra-*). A remission is observed when *Ra* > 0 (*Ra+* > *Ra-*). Immune-modulating drugs significantly reduce relapse frequency in relapsing-remitting multiple sclerosis by suppressing the immune attack, thereby lowering the *Ra-* value. Risk factors for MS include Epstein–Barr virus (EBV) seropositivity, low vitamin D levels, and obesity during adolescence (3). Each of these variables induces a pro-inflammatory reaction (59–61) that may trigger the onset of MS. In addition, the role of epitope spreading in the onset and relapse of multiple sclerosis is well-documented in experimental models and has also been observed in a prospective study of pediatric-onset CNS demyelinating diseases, specifically in patients who later developed MS (62, 63). Low sun exposure, leading to decreased vitamin D levels, and mononucleosis, may trigger MS relapses. Additionally, stress has been shown to induce relapses in MS patients (64). Brief periods of stress, lasting from minutes to hours, boost pro-inflammatory innate and adaptive immune responses (65). Referring back to Eq. 2, relapses (*Ra* **<** 0) may occur when brain local inflammation increases due to epitope spreading, temporary stress, temporary vitamin D depletion or EBV virion spread during the lytic phase of EBV life cycle, leading to demyelination and axonal injury. These effects are counteracted by remyelination and brain compensatory effects which may lead to remission (*Ra* > 0). Self-damping of inflammation may precede remyelination (66). Brain compensatory mechanisms result in improved motor function and physical ability typically observed during remission. This enhanced physical ability encourages increased physical activity, which is known to induce an anti-inflammatory effect (67). Accordingly, autoantibodies are detected in 86% of MS patients during the relapse phase, whereas only 30% of these patients test positive during remission (68). It is hypothesized that brain compensatory reserves diminish as MS progresses (57). A study involving MS patients supports this hypothesis (69). Assuming that brain reserves decline over time, the frequency of remissions must also decrease correspondingly. This, in turn, leads to a reduction in the frequency of relapses. When brain compensatory reserves reach a critical low level, tipping the balance in Eq. 2 towards a negative *Ra* value, RRMS converts into SPMS. Taken together, relapses in MS are a result of internal as well as environmental and recreational factors that induce immune reaction. The capacity of the CNS to regenerate or compensate for the loss of function is necessary for the induction of remission. When this capacity is lost, a period of continuous deterioration is established.

Reducing exposure to environmental factors that trigger MS could lower relapse frequency. This may be achieved through methods such as increasing sunlight exposure (3), vaccinating against EBV (70, 71), or managing stress (72). On the other hand, exercise, which is known to positively influence neural imaging outcomes and boost peripheral brain-derived neurotrophic factor (73), is believed to play a neuroprotective role or even a neuro-regenerative role in RRMS (74, 75). The author of this article suggests that, while exercise may improve fitness and quality of life throughout disease progression, any potential neuroprotective effect of exercise in MS is likely limited to the RRMS period, before brain functional reserves are exhausted and the compensatory response is lost.

## Unraveling the mystery of benign autoantibodies in healthy individuals

Many autoantibodies reported in cancer and AIDs are also observed in healthy individuals (76–80), evidently with no induction of AIDs. These autoantibodies will be referred to as common autoantibodies. One subset of these common autoantibodies is termed natural antibodies. Unlike adaptive antibodies, natural antibodies are produced by B1 lymphocytes (characterized by CD20+CD27+CD43+CD70− markers) and marginal-zone B cells (81). These natural antibodies do not undergo affinity maturation through antigen exposure or undergo extensive somatic hypermutation, and therefore exhibit low reactivity against self-antigens. Another group of common autoantibodies may originate from cross-reactive antibodies developed against infectious agent proteins, where the resemblance between foreign and self-peptides can stimulate the activation of self-reactive T or B cells (76, 82). Natural low affinity autoantibodies are mainly IgM, whereas the highly reactive group includes mainly IgG autoantibodies (83). While the reduced reactivity of natural autoantibodies may account for their low pathogenicity in triggering AIDs, it remains unclear why cross-reactive antibodies generated from exposure to external pathogens do not induce an autoimmune attack in otherwise healthy individuals. This article suggests that this benign reactivity may be due to a high recovery rate of the target tissue, which prevents symptom manifestation. In contrast, in AIDs with a low tissue recovery rate, symptoms can develop even at relatively low autoantibody levels, as damaged cells are not quickly replaced. Autoantibody levels associated with diseases with low recovery rate are expected to be low in healthy individuals (possibly below analytical detection limits), as higher levels would likely trigger symptoms. There are four AIDs that target tissues with low recovery rate after injury: advanced adult T1D, Hashimoto thyroiditis (HT), Graves’ disease (GD), and advanced primary biliary cholangitis (PBC). Contrary to all other AIDs, these diseases do not respond to corticosteroid or immunosuppressant treatments, as elucidated in an earlier publication (6). Although the liver has a high capacity of regeneration after injury, it is speculated that this property is lost due to extensive damage to hepatocytes following advanced PBC (6). As demonstrated in the former sections, these AIDs are not associated by cycles of remission and relapse. The following discussion will demonstrate that common autoantibodies targeting antigens associated with non-relapsing-remitting AIDs—such as T1D, GD, and PBC—are rare in the general population (though not every rare autoantibody in the general population is linked to non-relapsing-remitting AIDs). Additionally, it will be shown that common autoantibodies with high prevalence in the general population are associated with relapsing-remitting AIDs (though not all autoantibodies associated with relapsing-remitting AIDs are highly prevalent in the general population). Autoantibodies associated with Hashimoto’s thyroiditis exhibit low reactivity and therefore do not induce autoimmunity in either healthy individuals or HT patients; a high prevalent of these autoantibodies is not likely to trigger HT.

### Autoimmune diseases linked to autoantibodies rare in the general population

#### Type 1 diabetes

Autoantibodies associated with T1D have been described against five autoantigens: insulin, GAD65, insulinoma-associated antigen-2 (IA-2), zinc transporter 8 (ZnT8) and tetraspanin 7. Accumulating evidence suggests that GAD autoantibodies (GADA) are not pathogenic in T1D and may even play a protective role (84). As for the other T1D-related autoantigens:

1. Insulin - Using commercial kits, anti-insulin autoantibodies were detected in about 60% of 36 T1D patients but were not detected among any of 56 controls (healthy individuals and T2D patients) (85).
2. ZnT8: In a study arm that included a cohort of 70 adult healthy individuals, only one subject exceeded the positivity cut-off limit for ZnT8 (86). In another study, the prevalence of anti-ZnT8 autoantibodies in healthy adult Chinese subjects was 1% (4 out of 405) compared to 24% in T1D patients (130/539) (87). Among 128 patients with T2D, none was positive for ZnT8A (88).
3. IA-2: Among 128 adult Bulgarian patients with T2D, one (0.78%) tested positive for IA-2A (88). In another study, 9 out of 67 adult T1D patients were found to be positive for IA-2, while none of the 10 healthy controls tested positive for antibodies against this antigen (89). In a third study involving 761 healthy individuals under the age of 40 (used as controls), none tested positive for anti-IA-2 autoantibodies (90).
4. Tetraspanin 7: Autoantibodies against tetraspanin 7 were detected in 1 out of 94 non-diabetic subjects (91). Similarly, only 1 out of 52 non-diabetic subjects tested positive for tetraspanin 7 antibodies, although low levels (below the cutoff) were observed in other subjects (92).

Among a group of 101 children and adolescents without T1D (1**–**19 years old), only one (1%) was tested positive to anti-ZnT8 autoantibodies, one (1%) was tested positive to anti-insulin autoantibodies, and none was tested positive to anti-IA2 autoantibodies (93).

In summary, across all these studies, nearly all non-T1D individuals tested negative for autoantibodies against each of the four key T1D-related antigens.

#### Hashimoto’s thyroiditis

Patients with Hashimoto’s thyroiditis produce antibodies against various thyroid antigens, most commonly anti-thyroid peroxidase (anti-TPO) and antithyroglobulin (anti-Tg), with TSHR antibodies being less frequently detected (94). However, anti-Tg and anti-TPO antibodies do not directly cause thyroid cell destruction (95, 96). For this reason, autoantibodies against thyroid antigens do not trigger autoimmune reactions in either healthy individuals or HT patients. In HT, thyroid cell destruction is carried out by autoreactive T cells (26). Therefore, anti-TPO and anti-Tg antibodies may be observed in healthy individuals without triggering HT. Indeed, the prevalence of thyroid autoantibodies in the general population reported by Williams Textbook of Endocrinology (97) is 5% - 20% for anti-Tg and 8% - 27% for anti-TPO. A study in young asymptomatic females found a 5% prevalence for subjects positive only for anti-Tg antibodies, a 34% prevalence for those positive only for anti-TPO antibodies, and a 52% prevalence for subjects positive for both (98).

#### Graves’ disease

In Graves’ disease, the primary autoantibodies target the thyroid stimulating hormone receptor (TSHR) on thyroid cells. These autoantibodies stimulate the thyroid gland to produce excessive thyroid hormones, resulting in hyperthyroidism (29). In a study of 302 healthy subjects, none tested positive for TSHR stimulating or TSHR blocking antibodies (99).

#### Primary biliary cholangitis

Anti-mitochondrial antibodies (AMA), which target the E2 subunits of the 2-oxo acid dehydrogenase complexes (PDC-E2), serve as hallmark biomarkers for PBC found in 90-95% of affected patients. However, AMA is identified in less than 1% of healthy individuals (100).

Collectively, active autoantibodies that target tissues with limited recovery potential are linked to non-remitting-relapsing AIDs and are rarely detected in healthy individuals. Hashimoto’s thyroiditis is an exception, as its autoantibodies are largely inert, with autoreactivity primarily mediated by T cells.

### Autoimmune diseases linked to autoantibodies prevalent in the general population

In contrast to the rarity of common autoantibodies against antigens associated with non-remitting-relapsing AIDs in the general population, highly prevalent common autoantibodies in the general population are linked to remitting-relapsing AIDs (i.e., those involving tissues with high recovery capacity).

Some examples are provided below:

Shome et al. (76) identified 77 autoantibodies reported in cancer or AIDs that are also present in healthy individuals. This was achieved through protein microarray analysis, using data from healthy individuals screened against a set of human proteins. Antibodies against STMN4, ODF2, RBPJ, AMY2A, EPCAM, and ZNF688 showed the highest prevalence. Of these six antigens, three are associated with AIDs. STMN4 antibodies (47% prevalence in healthy individuals) are linked to primary Sjögren’s syndrome (101). Polymorphisms in RBPJ antibodies (37% prevalence in healthy individuals) are associated with rheumatoid arthritis (102), ZNF688 autoantibodies (29% prevalence in healthy individuals) are linked to systemic lupus erythematosus (103). AMY2A is associated with autoimmune pancreatitis; however, the high prevalence of AMY2 reported by Shome et al. contrasts with other published data (104) and shall not be discussed for this reason. Notably, primary Sjögren’s syndrome, rheumatoid arthritis, and systemic lupus erythematosus exhibit cycles of remission and relapse.

Antinuclear antibodies (ANA) are common in healthy individuals. Studies have shown that the prevalence of ANA associated with conditions like Sjögren’s syndrome, systemic sclerosis, systemic lupus erythematosus, rheumatoid arthritis, and soft tissue rheumatism exceeds 31.7% (78). All of these AIDs undergo cycles of remission and relapse.

With specific reference to remission-prone AIDs discussed in the previous section:

#### Alopecia areata

Autoantibodies targeting one or more hair follicle antigens, which were found in the sera of all 39 alopecia areata patients, were also detected in 12 out of 27 (44%) healthy controls (105). As discussed above, alopecia areata is a remitting-relapsing autoimmune disease.

#### Multiple sclerosis

Antibodies against native glycosylated myelin oligodendrocyte glycoprotein (MOG) were measured by ELISA in patients with MS and controls. Although median values in healthy controls were lower than those in MS patients, the differences were modest and varied depending on the specific antibody type (IgM or IgG) and the phase of MS (106). In another study, anti-MOG antibodies were detected in 65 out of 252 healthy individuals (26%) (107). In a study comparing autoantibodies to myelin basic protein (MBP) in healthy individuals and patients with MS, the sera from both study groups contained approximately equal amounts of MBP-reactive IgM (108). It appears that major antibodies against MS antigens—a disease with a remitting-relapsing phase in most patients—are highly prevalent in healthy populations.

#### Autoimmune pancreatitis

In one study, anti-smooth muscle antibodies (SMA) and anti-nuclear antibodies (ANA) were detected in 28% and 50% of AIP patients, respectively (109), while another study reported these antibodies in 17% and 76% of AIP patients, respectively (110). In healthy adults, high frequencies of ANA and SMA were observed, at 25% and 43%, respectively (111).

In conclusion, autoantibodies that target tissues with low regenerative capacity are associated with monophasic AIDs (i.e., AIDs that do not alternate between remissions and relapses) and are uncommon in the healthy population. In contrast, autoantibodies that target tissues with high regenerative potential are generally linked to relapsing-remitting AIDs and are highly prevalent in healthy individuals.

## Discussion

Phylogenetically ancient vertebrates, including amphibians and fish, possess the ability to regenerate significant portions of their bodies. In contrast, mammals exhibit only a limited capacity for regeneration (112). The human body consists of tissues with widely varying regeneration rates. While tissues such as the heart and central nervous system (CNS) exhibit little to no self-renewal, the liver and pancreas have a slow turnover, whereas the skin, blood, and intestine undergo active renewal (113, 114). The primary factor behind these differences in regenerative capacity is the varying abundance of stem cells across these tissues. Additionally, dedifferentiation—the process in which differentiated cells re-enter the cell cycle to generate new cells—may also contribute to tissue regeneration in mammals. For instance, the dedifferentiation of renal proximal tubular epithelial cells plays a role in kidney regeneration following acute kidney injury. Transdifferentiation is another mechanism of cell regeneration, which in mammalian tissues must be triggered by external stimuli (112). For example, cholangiocytes can transdifferentiate into hepatocytes following liver injury (115). These cell renewal mechanisms contribute to the differences among human tissues in their ability to replace lost cells.

As suggested above, a high recovery rate is a necessary condition for remission of chronic diseases including AIDs. Diseases that target tissues with low recovery rate like adult advanced T1D, GD, AGA and SPMS rarely improve spontaneously. Similarly, when the recovery capacity is impaired by the immune system, for example in PBC, remissions are very rare. On the other hand, AIDs that target organs with high recovery potential that is not impaired by the disease, like AA, AIP or RRMS, tend to develop cycles of remissions and relapses. It is suggested that brain compensatory effect is important in triggering RRMS remissions, and the wane of compensatory reserve drives the transition from RRMS to SPMS.

The collapse of immune privilege in anagen hair follicles is considered a primary cause of AA onset and relapse, while the restoration of immune privilege has been proposed to induce AA remission. IFN-γ has been shown to trigger immune privilege collapse, whereas molecules such as somatotropin, α-MSH, IGF-1, and TGF-β1 have been demonstrated to restore hair follicle immune privilege. This study proposes that IFN-γ, known to block Notch signaling and inhibit the differentiation of liver and bone marrow hematopoietic stem cells in adult mice, may also inhibit the differentiation of hair follicle stem cells. Similarly, IGF-1, known to enhance mesenchymal stem cell self-renewal and proliferation, possibly drives the proliferation of hair follicle stem cells. This suggests a new mechanism underlying relapses and remissions in AA in addition to HF-IP control.

Using Lewis rat model of experimental autoimmune uveitis (EAU), the group of Gerhild Wildner have shown that EAU can be either monophasic or remitting, depending on the autoantigen used for induction. Uveitis could be induced by immunization with retinal soluble antigen (S-Ag), interphotoreceptor retinoid-binding protein (IRBP) or their peptide derivatives (PDSAg from S-Ag and R14 from IRBP) in Complete Freund’s Adjuvant (CFA) as well as by the transfer of activated, antigen-specific T cells. EAU induced with PDSAg resulted in monophasic disease while immunization with peptide R14 led to remitting-relapsing disease. Similar effects were observed after the adoptive transfer of T cell lines specific for these peptides (116). The difference in the disease appearance (monophasic vs. remitting-relapsing) was attributed to “subtle differences in the T cell effector phenotype elicited” (5). The present work suggests a different explanation. The corneal epithelium is a self-renewing tissue sustained by stem cells located in the eye peripheral limbus. These limbal stem cells generate transient amplifying daughter cells, which renew the epithelium by migrating toward the center in a centripetal pattern (117). S-Ag (β arrestin-1) belongs to the family of arrestins which play a role in regulating stem cell characteristics, including self-renewal, in non-malignant stem cells (118). This may be also true with respect to IRBP since rat mesenchymal stem cells ameliorate EAU induced in rats by IRBP peptides (119, 120). If both S-Ag and IRBP antigens maintain ocular stem cell function, their attack by autoantibodies may impair ocular epithelium regeneration. Monophasic EAU following an attack by anti-SAg antibodies may be a result of a complete loss of ocular epithelial regenerative capacity, on the other hand, a remitting-relapsing disease following an autoimmune attack by anti-IRBP antibodies may be a consequence of a partial loss of this capacity.

The remission period in early T1D is widely discussed in the literature. The first hypothesis to explain this ‘honeymoon period’ was the concept of ‘β-cell rest.’ It proposed that exogenous insulin administration following T1D onset reduces the demand on β-cells, thereby improving insulin secretion and enhancing β-cell viability (121). Later it has been suggested that T1D is a cyclic relapsing–remitting disease, driven by the immune system. It has been proposed that factors contributing to the cyclic nature of T1D include epitope spreading, β-cell proliferation, and regulatory T cell activity (2). Epitope spreading refers to the spontaneous expansion of the autoimmune response to new epitopes (subdominant or cryptic epitopes) in an epitope-specific manner (122). A few points should be noted regarding this model: (1) The assumption of epitope spreading is strongly supported by data from T1D patients (123) and various other autoimmune conditions, such as experimental autoimmune encephalomyelitis (124), experimental autoimmune uveoretinitis (125), membranous lupus nephritis with anti-GBM antibodies (126), and autoimmune bullous diseases (127). (2) Although increased β-cell proliferation has been observed in NOD mice just before the onset of overt diabetes (128), to the best of the author’s knowledge, this has not been reported in humans, contrary to von Herrath et al. premise (2). However, restored insulin secretion, rather than increased β-cell proliferation, may be responsible for the remission observed in many T1D patients after disease onset. Supporting evidence for this assumption includes the finding that glucose-induced insulin secretion was restored in pancreatic tissues isolated from living subjects shortly after a T1D diagnosis, following several days in a non-diabetogenic environment (129).

**Table 1** summarizes the findings of this report.

**Table 1:** Autoimmune target tissue recovery rate, the tendency for a relapsing-remitting pattern, the likelihood of detecting autoantibodies in healthy individuals, and the response to immunosuppressive treatment.

Disease	Recovery rate	Remission(s)	Prevalence of autoantibodies in healthy individuals	Immunosuppressant treatment efficacy (6)
T1D (advanced)	low	no	low	low
AGA	low	no	not applicable (not an autoimmune disease)	low
GD (severe)	low	no	low	low
PBC	low	no	low	low
MS (SPMS)	low	no	high	low
HT	low in most patients/high in some patients (hypothesized)	no (most patients)/yes (some patients)	high (since autoantibodies are not reactive)	low/not available (high effectivity is hypothesized in patients with high recovery rate)
T1D (early)	high	yes	low	effective but transient
AA	high	yes	high	high
AIP	high	yes	high	high
MS (RRMS)	high	yes	high	high
GD (mild)	high	yes	low	Not available but high effectivity is hypothesized

T1D, Type 1 diabetes; AGA, Androgenic alopecia; GD, Graves’ disease; PBC, Primary biliary cholangitis; MS, Multiple sclerosis; HT, Hashimoto’s thyroiditis; AA, Alopecia areata; AIP, Autoimmune pancreatitis.

**Table 1** shows a correlation between target tissue recovery rate, the tendency for a relapsing-remitting pattern, the likelihood of detecting autoantibodies in healthy individuals, and the response to immunosuppressive treatment. AIDs targeting tissues with high recovery rates (due to high regenerative or compensatory capacities) tend to exhibit a relapsing-remitting pattern and respond to immunosuppressive treatments, including corticosteroids. Common autoantibodies highly prevalent in healthy individuals are often directed against target tissues with high recovery rates. In contrast, AIDs targeting tissues with low recovery rates do not display a relapsing-remitting pattern, do not respond to immunosuppressive treatments, and autoantibodies against their typical antigens are rarely found in healthy individuals.

**Figure 1:**
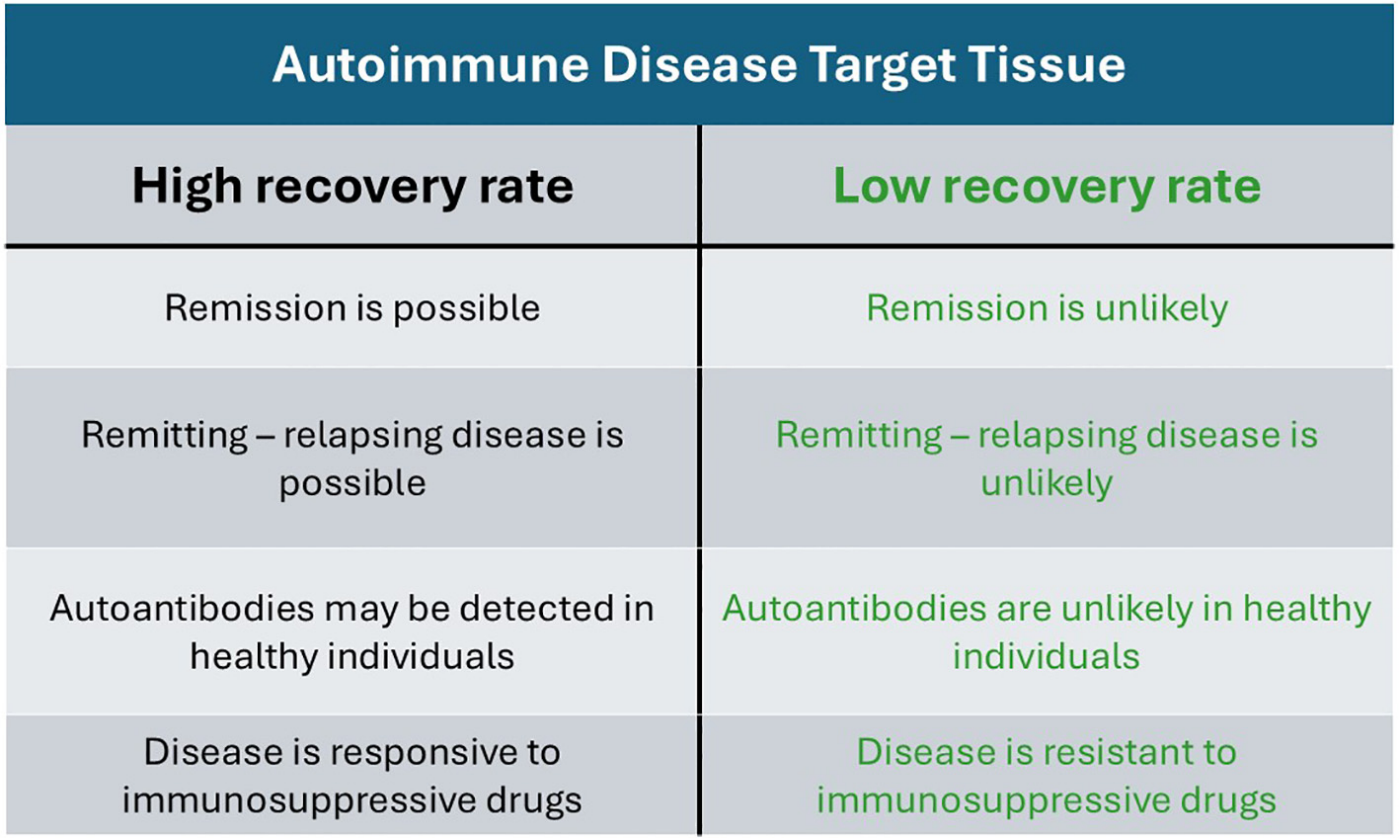
Comparative distinction between autoimmune diseases targeting tissues with high recovery rates and those targeting tissues with low recovery rates.

**Figure 1** illustrates the distinction between AIDs targeting tissues with a high recovery rate and those targeting tissues with low recovery rate.

## Summary

This report examines the role of target tissue recovery in autoimmune diseases, demonstrating that the rate of tissue recovery affects the likelihood of a relapsing-remitting pattern, the presence of autoantibodies in healthy individuals, and the response to immunosuppressive treatment. The enigma of harmless autoantibodies in healthy populations is unraveled. Analyzing the role of tissue recovery across various autoimmune diseases yields clinically significant insights. The use of immunosuppressive drugs in mild Graves’ disease and remitting Hashimoto’s thyroiditis is proposed. The decline in brain reserves is suggested as a key factor in the transition of multiple sclerosis from relapsing-remitting to secondary progressive disease. This decline limits the neuroprotective effect of exercise to the relapsing-remitting phase, before brain functional reserves are depleted. The remitting-relapsing pattern of alopecia areata, in contrast to the monophasic progressive pattern of androgenic alopecia, is explained. It is proposed that IGF-1, an agent that preserves immune privilege in anagen hair follicles in alopecia areata, also stimulates the regeneration rate of hair follicle cells. Similarly, IFN-γ, which disrupts hair follicle immune privilege, also slows the regeneration rate of hair follicle cells. This raises the possibility of a new mechanism that enables the remission-relapse cycles in alopecia areata. An explanation for the differing patterns of experimental autoimmune uveitis—either monophasic or remitting-relapsing—induced by immunization with two different antigens, is also provided, based on the rate of regeneration.

## Data Availability

The original contributions presented in the study are included in the article/supplementary material. Further inquiries can be directed to the corresponding author.
